# Osteoblast-Secreted Factors Promote Proliferation and Osteogenic Differentiation of Bone Marrow Stromal Cells via VEGF/Heme-Oxygenase-1 Pathway

**DOI:** 10.1371/journal.pone.0099946

**Published:** 2014-06-18

**Authors:** Lian-Fang Zhang, Jin Qi, GuiLai Zuo, Peng Jia, Xing Shen, Jin Shao, Hui Kang, HuiLin Yang, LianFu Deng

**Affiliations:** 1 Shanghai Key Laboratory for Bone and Joint Disease, Shanghai Institute of Traumatology and Orthopaedics, Ruijin Hospital, Shanghai Jiao Tong University School of Medicine, Shanghai, China; 2 Department of Orthopaedic, The First Affiliated Hospital of Soochow University, Suzhou, China; Georgia Regents University, United States of America

## Abstract

The hypoxia-inducible factors (HIFα) are the critical factors that couple angiogenesis and osteogenesis by activating transcription of VEGF in osteoblasts. Mice lacking von Hippel–Lindau gene (*Vhl*), thus overexpressing HIFα in osteoblasts develop extremely dense and highly vascularized long bones. Here we provide evidence that osteoblasts lacking *Vhl* overexpress and secrete high levels of VEGF, which subsequently promotes the proliferation and osteogenic differentiation of bone marrow stromal cells (BMSC) by promoting expression of Heme oxygenase-1 (HO-1) in BMSC. Conditioned medium from osteoblasts *Vhl* (CM-CRE) promoted the proliferation and osteogenic differentiation of BMSC, in comparison with conditioned medium derived from normal osteoblasts (CM-GFP). Recombinant VEGF stimulated the proliferation and osteogenic differentiation of BMSC culturing in CM-GFP. By contrast, VEGF-neutralizing antibody inhibited the proliferation and osteogenic differentiation of BMSC culturing in CM-CRE. Treatment with a HO-1 inhibitor, SnPP, significantly inhibited VEGF-induced BMSC proliferation and osteogenic differentiation. On the contrary, activation of HO-1 with CoPP reversed the suppressing of VEGF-antibody on the proliferation and osteogesis of BMSC culturing in CM-CRE. These studies suggest that osteoblasts promote the proliferation and osteogenic differentiation of BMCS by VEGF/HO-1 pathway.

## Introduction

The proper development and maintenance of bone size, shape, and integrity are based on communication among cells within the bone marrow microenvironment, such as osteoblasts, chondrocytes, osteocytes, osteoblasts and bone marrow mesenchymal stromal cells (BMSCs). BMSCs comprise a clonogenic, non-hematopoietic stem cell population that reside within the bone marrow stroma and is capable of differentiation into mesoderm-lineage cells e.g. osteoblasts, adipocytes and chondrocytes [1], [2]. BMSCs suppress osteoblast proliferation and transiently retard osteoblast differentiation by downregulating Runx2 [3]. However, the nature of communications between osteoblasts and BMSCs is still not clear.

Hypoxia-inducible factor (HIF) is one of the main coupling factors involved in the regulation of angiogenesis and osteogenesis during skeletal development and bone regeneration [4], [5]. Mice overexpressing HIFα in osteoblasts through selective deletion of the von Hippel-Lindau gene (Vhl) expressed high levels of VEGF and developed extremely dense, heavily vascularized long bones. However, loss of Vhl and upregulation of HIFα in osteoblasts have minimal effects on in vitro osteoblast proliferation, survival, and differentiation [5].

Heme oxygenase-1 (HO-1) is the rate-limiting enzyme in heme degradation, catalyzing the cleavage of the heme ring to form ferrous iron, carbon monoxide (CO) and biliverdin [6], [7]. HO-1 has strong implications in bone marrow stem cell differentiation [8], [9]. Recent studies have shown that VEGF may activate the expression of HO-1 [10], [11], and HO-1 expression is increased during osteoblast stem cell development [12]. Furthermore, overexpression of HO-1 increases human osteoblast stem cell differentiation [13].

We therefore hypothesized that VEGF synthesized and secreted by osteoblasts may induce the expression of HO-1 in BMSCs, and promote their proliferation and differentiation. In the present study, we tested the effect of conditioned medium from Vhl gene defect osteoblasts on the proliferation and differentiation of BMSC, and examined whether VEGF and HO-1 are involved in it.

## Materials and Methods

### Animals

Ethics Statement: All procedures involving mice were approved by the Shanghai Jiaotong University Animal Study Committee and were carried out in accordance with the guide for the humane use and care of laboratory animals.

Osteoblast Vhl conditional knockout (CKO) mice were generated by intercrossing OC-Cre transgenic mice with mice containing Vhl floxed allele (Vhl^flox/flox^) (both mice kindly provided by Dr. Thomas L. Clemens, Department of Orthopaedic Surgery, Johns Hopkins University School of Medicine, Baltimore, MD). Littermates were used as controls for all experiments. PCR of DNA isolated from tail biopsies was used to confirm genotypes as described previously [5].

### Skeletal Phenotyping and Histological Analysis

MicroCT (GE Locus SP) was used to access the bone mass, density, geometry, and trabecular microarchitecture of the right femurs from 6-week-old control and condition knockout (CKO) mice. Parameters computed from these data include trabecular thickness, number, separation, and connectivity at the distal femoral metaphysis and cortical thickness and cross-sectional area at the mid-diaphysis. The left femurs were fixed in 4% paraformaldehyde, decalcified in 10% EDTA, paraffin embedded, and stained with H&E using standard methods. For immunohistochemistry, antigen retrieval was performed by boiling in 10 mM sodium citrate (pH 6.0) for 5 minutes. Sections were incubated with antibodies against HIF-1α (Abcam), HIF-2α (Abcam), VEGF (Novus Biologicals), PCNA (R&D Systems), and HO-1 (Abcam). The slides were examined using a Zeiss Axio microscope. Image-Pro Plus software was used to quantify the integrated optical density.

### Collection of Conditioned Media from Primary Osteoblast with and without Vhl

Osteoblasts were isolated from calvaria of newborn Vhl^flox/flox^ mice by serial digestion in 1.8 mg/ml collagenase type I (Sigma) solution. Calvaria were digested in 10 ml of digestion solution for 15 min at 37°C with constant agitation. The digestion solution was collected, and digestion was repeated with fresh digestion solution an additional four times. Digestions three to five (containing the osteoblasts) were pooled together, centrifuged, washed with α-minimal essential medium (αMEM) containing 10% FBS, 1% penicillin/streptomycin and plated overnight at 37°C in a humidified incubator supplied with 5% CO_2_. To disrupt Vhl in vitro, osteoblasts were grown to approximately 70% confluence and then infected with control adenovirus expressing green-fluorescent protein (Ad-GFP) or adenovirus expressing Cre recombinase (Ad-Cre, Vector Biolabs) at an MOI of 100. Osteoblasts were harvested 48 h after adenoviral infection and deletion efficiency was assessed in a portion of the cell population by real-time PCR and Western Blot. The remaining cells were replated on 100-mm tissue culture plates and subjected to preparation.

Mouse primary osteoblasts were plated on 100-mm plates with the density of 10^6^ cells/plate, and cultured in α-MEM supplemented with 10% FBS and 1% penicillin/streptomycin. The cells cultured for 3, 5 and 7 days in complete media and were then cultured with DMEM without serum or penicillin/streptomycin for 12 hours before collecting the conditioned media (CM) from control (CM-GFP) and Vhl-deficient (CM-CRE) osteoblasts. Immediately after collection, the CM was centrifuged at 1,200 rpm for 5 min, and stored at −80°C.

### Enzyme-Linked Immunosorbent Assay (ELISA) for VEGF

By using a specific ELISA test, according to the manufacturer’s recommendation, we evaluated the level of VEGF (R&D Systems) in the culture supernatant of osteoblasts.

### Isolation, Culture, and Expansion of Bone Marrow Mesenchymal Stromal Cells

BMSCs were isolated from Vhl^flox/flox^ mice femoral and tibial bone marrow (BM) as previously described [14], [15]. Briefly, muscles and the entire connective tissue were detached, and the epiphyses were removed. Marrow was harvested by inserting an 27-gauge syringe needle into one end of the bone shaft and flushing the contents into a 60-mm culture dish containing proliferation culture medium, consisting of complete medium–Dulbecco’s modified Eagle’s medium-low glucose (DMEM) (Invitrogen) supplemented with 20% (v/v) screened fetal bovine serum (FBS) (Invitrogen). The cell suspension was filtered by a 70-mm filter mesh to remove any bone spicules or muscle and cell clumps. Filtered BM cells were cultured in 100-mm culture dishes in 10 ml of complete medium at a density of 107 cells ml-1. The plates were incubated at 37°C with 5% CO_2_ in a humidified chamber without disturbing them. After 3 h, the nonadherent cells that accumulate on the surface of the dish were removed by changing the medium and replacing with fresh complete medium. After 14 days, the cells had grown to 90% confluence and were harvested by trypsinization and passaged at 1∶3.

### BMSC Proliferation Assay

The inhibition of HO-1 enzymatic activity by tin-protoporphyrin-IX (SnPP) and induction of the HO-1 by cobalt protoporphyrin (CoPP) have been used in our investigation [16]–[18].

The effect of CMs on BMSC proliferation was evaluated with CCK8 assay (Beyotime institute of biotechnology), according to the manufacturer’s instructions. Briefly, 10^3^ BMSCs (passage 6) were seeded in a volume of 100 µl into each well of six 96-well plates. Six kinds of CMs were used: i) 1 part CM-GFP: 1 part DMEM+20% FBS; ii) 1 part CM-GFP+VEGF (25 ng/ml, R&D Systems): 1 part DMEM+20% FBS; iii) 1 part CM-GFP+VEGF (25 ng/ml, R&D Systems) + SnPP (20 µM, Frontier Scientific Inc.): 1 part DMEM+20% FBS; iv) 1 part CM-CRE: 1 part DMEM+20% FBS; v) 1 part CM-GFP+VEGF-antibody (100 ng/ml, Novus Biologicals): 1 part DMEM+20% FBS; vi) 1 part CM-GFP+VEGF-antibody (100 ng/ml, Novus Biologicals) + CoPP (25 µM, Frontier Scientific Inc.): 1 part DMEM+20% FBS. All assays were performed in quadruplicate. After various incubation periods ranging from one to seven days, cells were incubated with the yellow CCK8 solution (10 µl) for approximately 2 h, and the absorbance was finally determined at 450 nm using a micro plate reader.

### BMSC Differentiation Assay

For osteogenic differentiation, BMSCs are cultured in above six CMs containing 10^−7^ M dexamethasone (Sigma-Aldrich), 10 mM β-glycerol phosphate (Sigma-Aldrich) and 50 µM ascorbate-2-phosphate (Sigma-Aldrich) [19]–[23]. And for adipogenic differentiation, BMSCs are incubated in CMs containing 10^−6^ M dexamethasone, 0.5 µM isobutylmethylxanthine (IBMX) (Sigma-Aldrich) and 10 ng/ml insulin (Sigma-Aldrich) [19], [21], [22], [24]. Cells were seeded into 60-mm culture dish, and medium was exchanged every 3 days for 21 days.

Bone mineralization was determined using Alizarin Red S (Sigma-Aldrich, St. Louis, MO) staining and phase-contrast microscopy 21 days after treatment. Cell were incubated with 2% alizarin red at pH 4.2 for 10 min and subsequently washed with distilled water. Subcultured cells were examined by phase-contrast microscopy at 21 days to determine cell morphology and to verify the presence of mineralized nodules.

### Quantitative Real-Time PCR

Total RNA was extracted from osteoblasts and BMSCs using the Trizol method (Invitrogen). One microgram of pure RNA, as assessed spectrophotometrically using the A260/A280 ratio, was reverse transcribed using the iScript cDNA synthesis system (Bio-Rad). Two microliters of cDNA was then subjected to PCR amplification using iQ SYBR Green Supermix (Bio-Rad) and sequence-specific primer pairs in an Opticon Continuous Fluorescent Detector (MJ Research). The primers used are listed in Table 1.

**Table 1:** Primer sequences used for real time PCR analysis.

Gene	Forward (5′→3′)	Reverse (5′→3′)
Vhl	GCCTATTTTTGCCAACATCACA′	TCATTCTCTCTATGTGCTGGCTTT
Hif1α	CAAGATCTCGGCGAAGCAA	GGTGAGCCTCATAACAGAAGCTTT
Hif2α	CAACCTGCAGCCTCAGTGTATC	CACCACGTCGTTCTTCTCGAT
Vegf	CCACGTCAGAGAGCAACATCA	TCATTCTCTCTATGTGCTGGCTTT
Runx2	ATGCTTCATTCGCCTCAC	CTCACGTCGCTCATCTTG
OC	TCTGCTCACTCTGCTGAC	GGAGCTGCTGTGACATCC
Osterix	ATGGCGTCCTCTCTGCTTG	TGAAAGGTCAGCGTATGGCTT
PPARγ	TCGCTGATGCACTGCCTATG	GAGAGGTCCACAGAGCTGATT
C/EBPα	CAAGAACAGCAACGAGTACCG	GTCACTGGTCAACTCCAGCAC
HO-1	AAGCCGAGAATGCTGAGTTCA	GCCGTGTAGATATGGTACAAGGA
β-Actin	CCCAGAGCAAGAGAGG	GTCCAGACGCAGGATG

### Western Blot Analysis

Nuclear extracts (50 µg) were boiled for 5 min in Laemmli buffer [62.5 mM Tris (pH 6.8), 1% SDS, 20% glycerol, 0.01% bromophenol blue, and 100 mM DTT] and separated on 6% SDS-PAGE gels. Gels were then transferred to 0.2 AM nitrocellulose membranes. After blocking with TBS-T [TBS (pH 7.4) and 0.1% Tween-20] containing 5% low fat milk, the membranes were incubated with primary antibody overnight in blocking buffer followed by horseradish-peroxidase-conjugated secondary antibody for 2 hr and developed by enhanced chemiluminescence (Amersham Pharmacia Biotech, Piscataway). Analysis of HIF-1α and HIF-2α was performed on evenly loaded immunoblots by sequential reprobing with each antibody after stripping using 2% SDS, 62.5 mM Tris pH 6.7, 100 mM mercaptoethanol for 30 min at 50-C. Antibodies against HIF-1α, HIF-2α and HO-1 were all from Abcams.

### Statistical Analysis

All data are presented as the mean ± standard deviation (SD). Statistical analysis was performed with SPSS (v19.0) using Student’s t-test or one-way ANOVA. A P-value of <0.05 was considered significant.

## Results

### Osteoblasts Lacking Vhl Promote the Proliferation of BMSCs through Secreting a Large Number of VEGF

Micro-CTs of femurs showed a dramatic increase in bone volume in CKO mice femurs compared with wild-type (WT) controls at 3 weeks and 6 weeks of age (**Fig. S1A**). Quantitative analysis revealed the bone mineral density (BMD) of femoral distal metaphyseal trabecular bone was significantly increased at 3 weeks (p = 0.0292) and 6 weeks (p<0.0001) of age compared with the control mice (**Fig. S1B**). In contrast, the BMD of middle femur cortical bone was decreased at 3 weeks (p = 0.0012) and 6 weeks (p = 0.0380) of age compared with the control mice (**Fig. S1C**). Consistent with these findings, HE staining showed significantly more new trabecular bone formation in the CKO mice, as compared with the control mice (**Fig. S1D**).

Immunohistochemistry on sections of femurs indicated that HIF-1α, HIF-2α and VEGF expression in osteoblasts lining bone surfaces were all markedly higher in CKO mice than those in WT animals which were expressed at a relatively low level (**Fig. S2A, S2B**). To test the relative gene-expressions in-vitro, we knocked out Vhl in osteoblasts with Ad-Cre. The mRNA levels of Hif-1α and Hif-2α did not change; however, their proteins levels were increased indicating that Vhl regulated HIFs at the post-transcriptional level (**Fig. S2C, S2D**). Real-time PCR and ELISA showed that osteoblasts lacking Vhl synthesized and secreted increasing amounts of VEGF (****).

**Figure 1:**
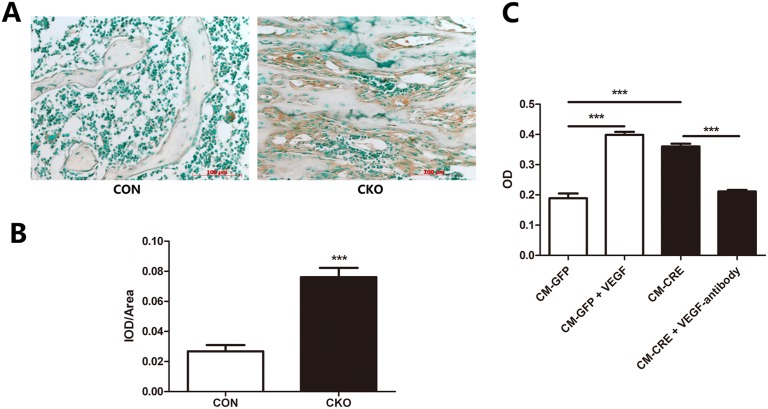
Osteoblasts lacking Vhl promote BMSC proliferation. (A) Representative histological sections of distal femurs from 3-week-old OC-Cre: Vhl^flox/flox^ (CKO) and littermate control (CON) mice after staining with antibodies against PCNA as described in Methods. Sections were counterstained with methyl green. Original magnification, ×200. (B) Quantitative analysis of A. (C) CCK-8 test of BMSCs cultured with CMs. Data represent mean ± SD. *,p<0.05; **,p<0.01, ***,p<0.001.

To investigate the proliferation of BMSCs after the Vhl gene was specifically deleted in CKO mice osteoblasts as they expressed osteocalcin, we used immunohistochemistry to detect the expression of PCNA in bone tissue. We found that the expression of PCNA was significantly increased in bone marrow cells surrounding trabecular bone of CKO mice (**Fig. 1A, 1B**). To investigate the effect of lack of Vhl in osteoblasts on the proliferation of BMSCs in vitro, we collected the supernatants of osteoblasts infected with Ad-GFP or Ad-Cre, and collected the resulting conditional mediums (CM-GFP and CM-CRE, respectively). BMSCs cultured in CM-GFP exhibited a small degree of proliferation, which was greatly enhanced by treatment with a recombinant VEGF. By contrast, proliferation of BMSCs cultured in CM-CRE was much greater than that of controls and was virtually abolished by pre-incubation with a VEGF-neutralizing antibody (**Fig. 1C**). This finding suggests that VEGF is upregulated in the long bones of the Vhl CKO mice and contributes to increased proliferation of BMSCs in bone tissue.

### CM from Osteoblasts Lacking Vhl Increases BMSC-derived Osteoblast Differentiation

**Figure 2:**
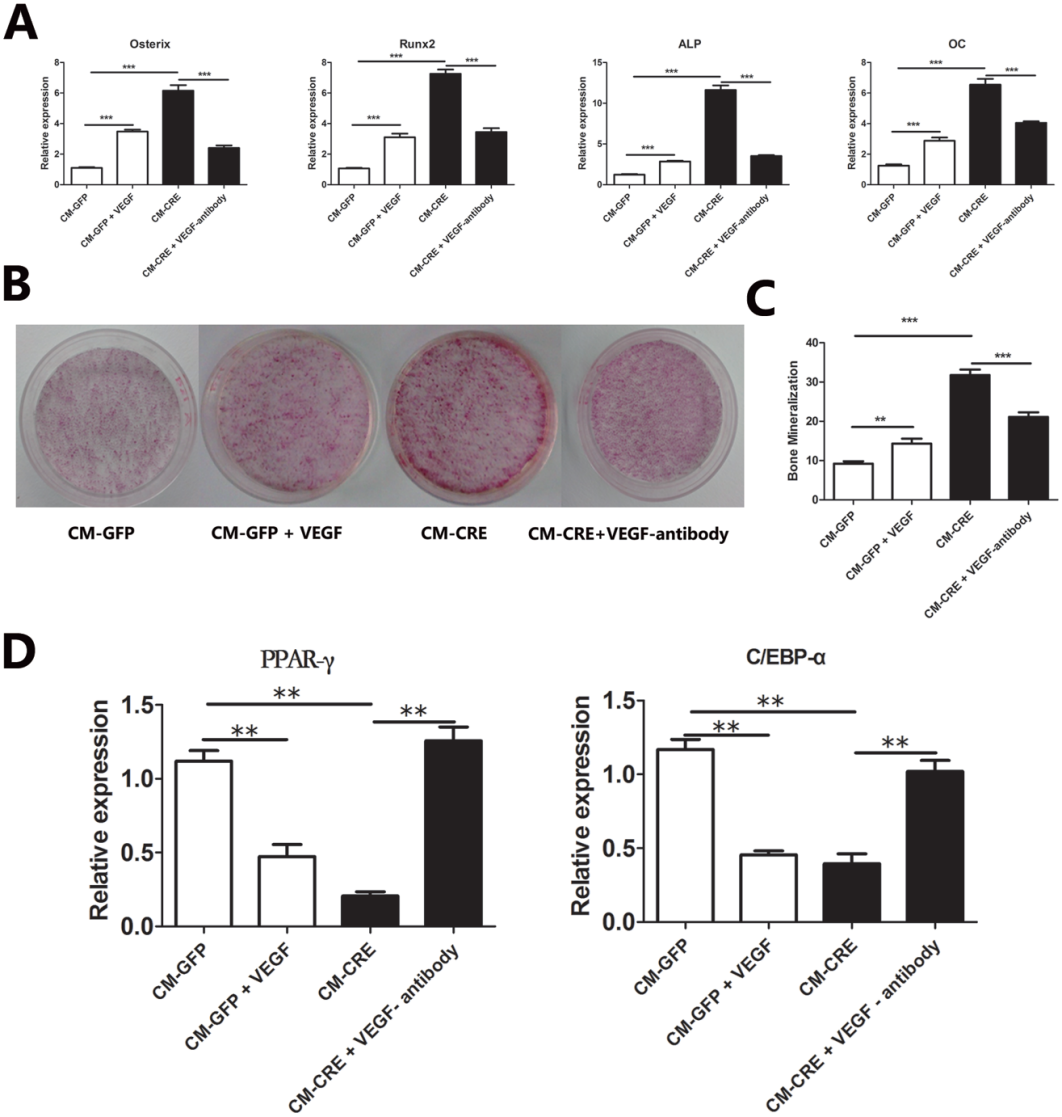
CM from osteoblasts lacking Vhl increases BMSC-derived osteoblast differentiation,and decreases BMSC-derived adipogenesis. (A) Measurement of osterix, Runx2, ALP and OC mRNA expression by quantitative real-time PCR at day 14 of osteogenic induction. (B) Alizarin Red S (Sigma-Aldrich, St. Louis, MO) staining of BMSCs cultured with CMs at day 21 of osteogenic induction. (C) Numbering of mineralized nodules of B. (D) The mRNA level of PPAR-γ and C/EBP-α of BMSCs at day 14 of adipogenic differentiation. Data represent mean ± SD. *,p<0.05; **,p<0.01, ***,p<0.001.

Addition of conditioned media from CM-CRE cells onto BMSCs undergoing osteogenic differentiation significantly increased osteoblastic markers as compared to conditioned media from CM-GFP cells as assessed by quantitative PCR for osterix, RUNX-2, osteocalcin and ALP (**Fig. 2A**). The addition of recombinant VEGF promoted osteoblast differentiation of BMSCs cultured with conditioned media from CM-GFP. On the contrary, osteoblast differentiation of BMSCs cultured in conditioned media from CM-CRE was suppressed after pre-incubating with a VEGF-neutralizing antibody as assessed by quantitative PCR (**Fig. 2A**). Similar results were obtained by Alizarin Red staining and quantification of numbers of calcium nodules (**Fig. 2B, C**).

### CM from Osteoblasts Lacking Vhl Decreases BMSC-derived Adipogenesis

The expressions of PPAR-γ and C/EBP-α during differentiation were significantly decreased when BMSCs were cultured with the conditioned media from CM-CRE at 14 days compared with the conditioned media from control (CM-GFP) (**Fig. 2D**). The addition of recombinant VEGF suppressed the adipogenesis of BMSCs cultured in the conditioned media from CM-GFP. Adipogenesis of BMSCs cultured in the conditioned media from CM-CRE was promoted after pre-incubation with a VEGF-neutralizing antibody (**Fig. 2D**). These findings suggest that VEGF secreted by Vhl-deficient osteoblasts can promote BMSC-derived osteoblast differentiation and suppress BMSC-derived adipogenesis.

### Osteoblasts Lacking Vhl Induced the Expression of HO-1 in BMSCs through VEGF

**Figure 3:**
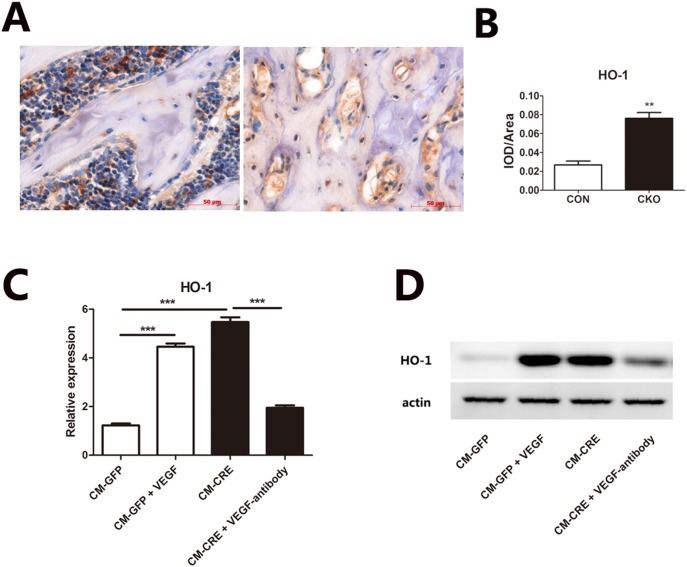
Osteoblasts lacking Vhl induced the expression of HO-1 in BMSC through VEGF. (A and B) Immunohistochemistry of HO-1 in bone marrow cells of distal femurs from 3-week-old OC-Cre: Vhl^flox/flox^ (CKO) and littermate control (CON) mice. Original magnification, ×200. (C) Total mRNA was extracted from BMSCs cultured with CMs for 3 days, and gene expression for HO-1 was determined by quantitative real-time PCR. (D) Western blot of HO-1 of BMSCs cultured with CMs for 3 days. Data represent mean ± SD. *,p<0.05; **,p<0.01, ***,p<0.001.

To investigate the mechanism of VEGF regulating the differentiation of BMSCs, we first detected the HO-1 expression in CKO and CON mice bone. Immunohistochemistry detected that the expression of HO-1 was significantly increased in bone marrow cells surrounding trabecular bone of CKO mice than in WT mice (**Fig. 3A, 3B**). Quantitative PCR revealed that the HO-1 mRNA level of BMSCs cultured in the conditioned media from CM-CRE was much higher than the cultures in the conditioned media from CM-GFP (**Fig. 3C**). Similar trend was observed at the protein level as assessed by immunoblotting (**Fig. 3D**). Incubation of the CM-GFP and CM-CRE with recombinant VEGF or a VEGF-neutralizing antibody respectively reversed the effects of the conditioned media on HO-1 expression (**Fig. 3C, 3D**).

### VEGF Promotes the Proliferation and Osteogenic Differentiation of BMSCs by Increasing their Expression of HO-1

**Figure 4:**
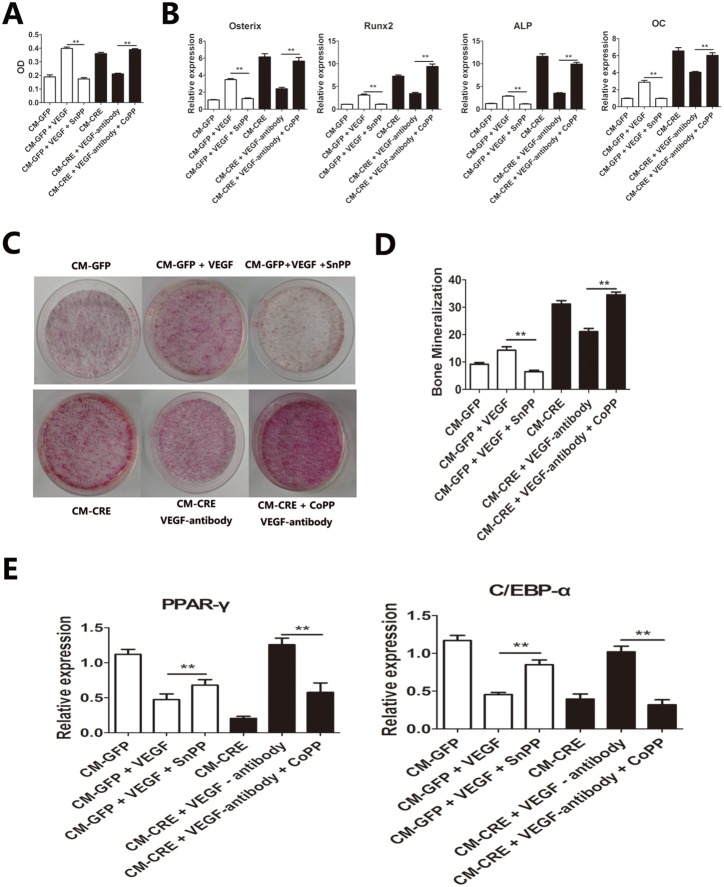
VEGF promotes the proliferation and osteogenic differentiation of BMSC through increasing the expression of HO-1 in BMSC. The proliferation and differentiation of BMSC cultured with CMs after the inducing or inhibiting of HO-1. (A) CCK-8 test the proliferation of BMSCs. (B C D) Osteogenic differentiation of BMSCs. (E) Adipogenic differentiation of BMSCs. Data represent mean ± SD. *,p<0.05; **,p<0.01, ***,p<0.001.

To evaluate the effect of HO-1 blockade or activation, SnPP (20 µM) and CoPP (25 µM) were added to the conditioned media. The presence of the HO-1 inhibitor SnPP significantly inhibited VEGF-induced BMSC proliferation, whereas induction of HO-1 with CoPP resulted in BMSC proliferation, which was suppressed by VEGF-antibody (**Fig. 4A**). Furthermore, blockade of HO-1 inhibits VEGF-induced in vitro angiogenesis, which was confirmed by the decreased expression of osteogenic differentiation marker genes (**Fig. 4B**) and the reduction of calcium nodules (**Fig. 4C, 4D**). Conversely, induction of HO-1 with CoPP reversed the VEGF-antibody inhibition of BMSC osteogenic differentiation when cultured in the CM-CRE (**Fig. 4B, 4C, 4D**).

The blockade of HO-1 was accompanied by a significant increase in the levels of PPAR-γ and C/EBP-α of BMSCs cultured in the CM-GFP with VEGF (**Fig. 4E**). The induction of HO-1 inhibited the adipogenesis of BMSCs cultured in the CM-CRE and that had been pre-incubated with a VEGF-neutralizing antibody (**Fig. 4E**). These findings suggest that VEGF promotes the proliferation and osteogenic differentiation of BMSCs through increasing their expression of HO-1.

**Figure 5:**
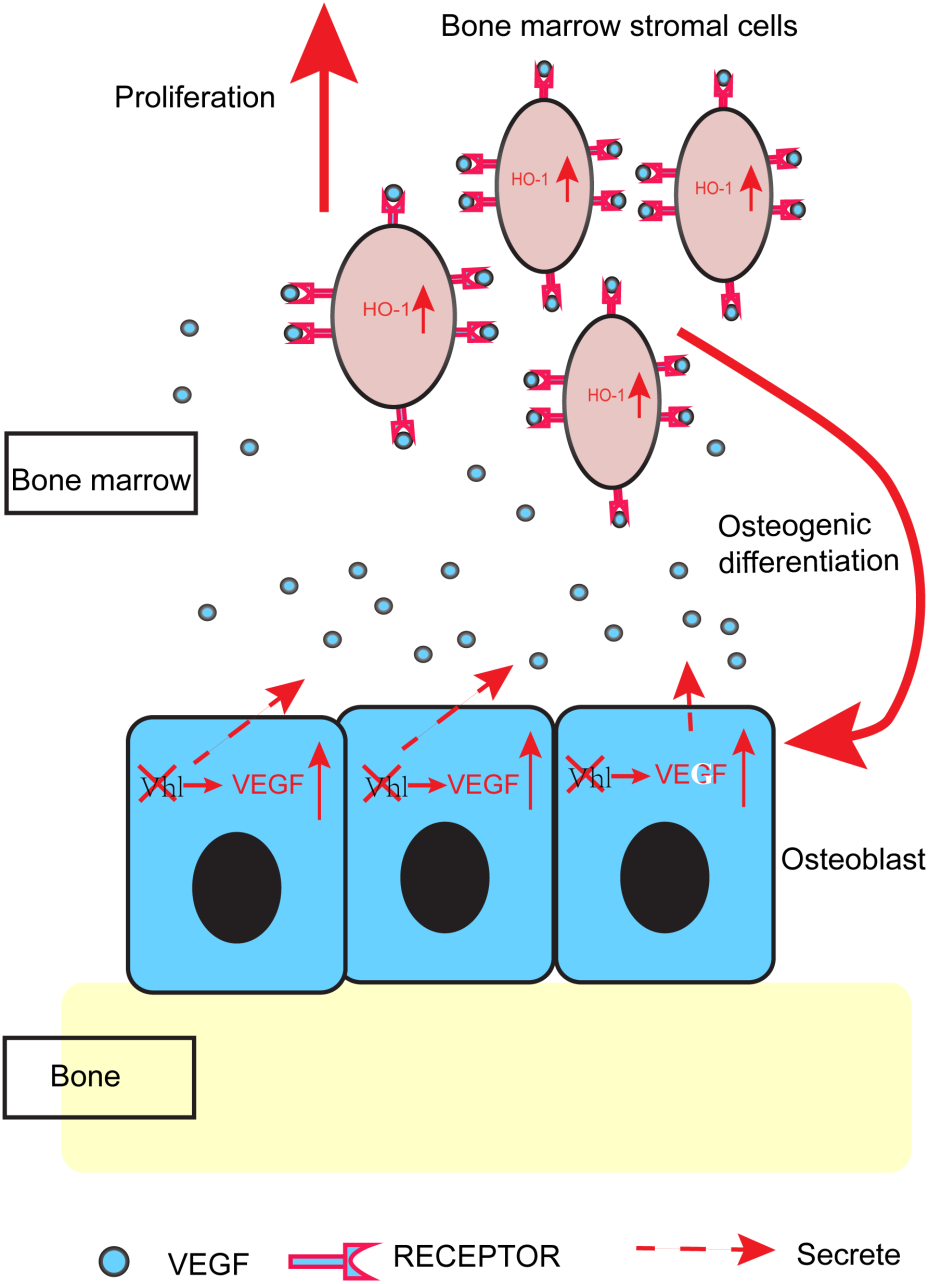
Model for the crosstalk of the crosstalk of osteoblasts and BMSCs. Osteoblasts lacking Vhl overexpress and secret high levels of VEGF, which subsequently promotes the proliferation and osteogenic differentiation of BMSCs by promoting expression of HO-1 in BMSCs.

Therefore, we can draw a conclusion that osteoblasts lacking Vhl overexpress and secret high levels of VEGF, which subsequently promotes the proliferation and osteogenic differentiation of BMSCs by promoting expression of HO-1 in BMSCs (**Fig. 5**).

## Discussion

It is well known that VEGF is actively responsible for hypertrophic cartilage neovascularization through a paracrine release by chondrocytes [25], and couples hypertrophic cartilage remodeling, ossification and angiogenesis during endochondral bone formation [26]. VEGF-A gene transfer significantly increased bone formation parameters, such as osteoblast number, osteoid volume, and bone volume, especially in trabecular bone [27]. In the present study, we show that mice lacking the Vhl gene in osteoblasts develop extremely dense heavily vascularized trabecular bone. Disruption of Vhl had no appreciable effect on ALP expression and only slightly increased calcified nodule formation. Moreover, the expression of runt-related transcription factor 2 (Runx2) and OC, markers for early and late osteoblast differentiation, respectively, was not significantly altered in the Vhl-deficient cells [28]. However, the number of osteoclasts expressed either as number per bone surface or number per total tissue area was not significantly different from that of controls at 3 weeks [28]. Therefore, we considered BMSC performance to be the direct reason for excessive bone formation in Vhl CKO mice.

In mice, the mobilization and recruitment of BMSC is dependent upon the activity of VEGF receptor 1 (VEGFR1) encoded by Flt1 [29]. In contrast, VEGFR2, which is encoded by the Flk1 gene in mice is required for BMSC survival, proliferation, and differentiation [30], [31]. In spite of the absence of VEGF receptors in human adult mesenchymal stem cells, VEGF-A can stimulate platelet-derived growth factor receptors (PDGFRs), thereby regulating BMSC migration and proliferation [32]. Previous investigation confirmed that VEGF is potent in promoting BMSC proliferation, and facilitates bone morphogenetic protein (BMP4) [33] and BMP7 [34] mediated BMSC osteogenesis through multiple mechanisms. Inhibition of VEGF synthesis and function by antisense oligonucleotide and by suramin, respectively arrested the BMP-7 induced alkaline phosphatase activity and mineralized bone nodule formation [35]. VEGF-A enhances BMSC proliferation by increasing phospho-Akt, phospho-ERK-1/2 and phospho-PKC [36]. We found that VEGF existing in the CM-CRE can promote the proliferation of BMSC, and upregulate osterix, Runx2, ALP and osteocalcin mRNA level, and downregulate the PPAR-γ and C/EBP-α mRNA levels in BMSCs resulting in activation of osteogenesis and repression of adipogenesis. Osteoblasts lacking the Vhl gene can produce and secrete a large number of VEGF, and promote proliferation and osteoblast differentiation.

Previous studies have shown that human VEGF165 could activate Nrf2 in an ERK1/2-dependent manner and HO-1 expression was up-regulated by Nrf2 in Human choriocarcinoma BeWo cells [10]. An increase in HO-1 expression following exposure to VEGF was seen at 24 hours and was maximal at 48 hours in human umbilical vein endothelial cells (HUVECs), the human microvascular endothelial cell 1 (HMEC-1), and bovine aortic ECs [37]. First, we found that deleting the Vhl gene in osteoblasts hindered the degradation of HIFα and lead to the increase in synthesis and secreting of VEGF both in vivo and in vitro. Then, we confirmed that CM-CRE induced the expression of HO-1 in BMSCs that were inhibited by VEGF-antibody. On the contrary, recombinant VEGF increased the mRNA and protein levels of HO-1 in BMSCs cultured in CM-GFP. These findings indicate that osteoblasts may induce the expression of HO-1 in BMSCs by VEGF in paracrine manner.

HO-1 expression is increased during osteoblast stem cell development, and the increase in HO-1 expression precedes an increase in alkaline phosphatase, bone morphogenic protein, osteonectin, and RUNX-2 mRNA [12]. Whether BMSCs differentiate into osteoblasts or adipocytes is due to multiple signaling pathways including those heavily influenced by HO-1 and -2 [13]. The OGP-mediated increase in HO-1 levels increases osteoblast proliferation and differentiation and is associated with an increase in osteoblast function, via an increase in AKT, pAKT, eNOS and p-eNOS [12]. Past research has demonstrated that eNOS is an enzyme expressed in osteoblasts that, when deficient, has been shown to lead to a significant reduction in bone formation in murine models [38]. Both eNOS and NO are stimulators of BMP-2 and increase the differentiation of osteoblasts [39], [40]. In our investigation, chemical inhibition of HO-1 enzymatic activity by SnPP, impaired VEGF-induced proliferation and differentiation of BMSCs cultured in CM-GFP. On the contrary, CoPP reversed the suppression of VEGF-antibody on the proliferation and differentiation of BMSCs cultured in CM-CRE by inducing of HO-1 enzymatic activity.

In this paper, we demonstrated that osteoblasts may have a significant effect on promoting BMSC proliferation and osteogenic differentiation. These results provide a broader understanding of the role of the hypoxia-inducible factor pathway in the crosstalk between osteoblast and BMSCs.

## Supporting Information

Figure S1 **Overproduced trabecular bone in Vhl CKO mice.** (A) Representative µCT images of the femurs from OC-Cre: Vhl^flox/flox^ (CKO) and littermate control (CON) mice at 3 and 6 weeks of age. Scale bars: 1.0 mm. (B) BMD of femoral distal metaphyseal trabecular bone of CON and CKO mice at the age of 3 and 6 weeks. (C) BMD of BMD of middle femur cortical bone of CON and CKO mice at the age of 3 and 6 weeks. Data represent mean ± SD. *,p<0.05; **,p<0.01. (D) H&E-stained longitudinal sections of distal femur and cross sections of middle femur from CON and CKO mice at the age of 3 and 6 weeks. Original magnification, ×100.
(TIF)

Figure S2 **Osteoblasts losing Vhl overexpressed Hif-1α, Hif-2α and Vegf in vivo and in vitro.** (A) HIF-1α, HIF-2α, and VEGF protein detection in the distal femoral metaphysis of 3-week-old CON and CKO mice by immunohistochemistry. Original magnification, ×200. (B) Quantitative analysis of A. (C) Quantitative real-time PCR analysis was performed in osteoblasts 48 hours after adenoviral infection. (D) Western blot analysis of HIF-1α, and HIF-2α in osteoblasts. (E) ELISA assay of VEGF (R&D Systems) in the culture supernatant of osteoblasts 3, 5 and 7 days after adenoviral infection. White bars represent Ad-GFP infection; black bars represent Ad-CRE infection. Data represent mean ± SD. *,p<0.05; **,p<0.01, ***,p<0.001.
(TIF)
