# Supplementary figures and images for: Osteoblast-Secreted Factors Promote Proliferation and Osteogenic Differentiation of Bone Marrow Stromal Cells via VEGF/Heme-Oxygenase-1 Pathway
Source: PLoS One. 2014 Jun 18;9(6):e99946. doi: 10.1371/journal.pone.0099946 (PMC4062480; doi:10.1371/journal.pone.0099946)

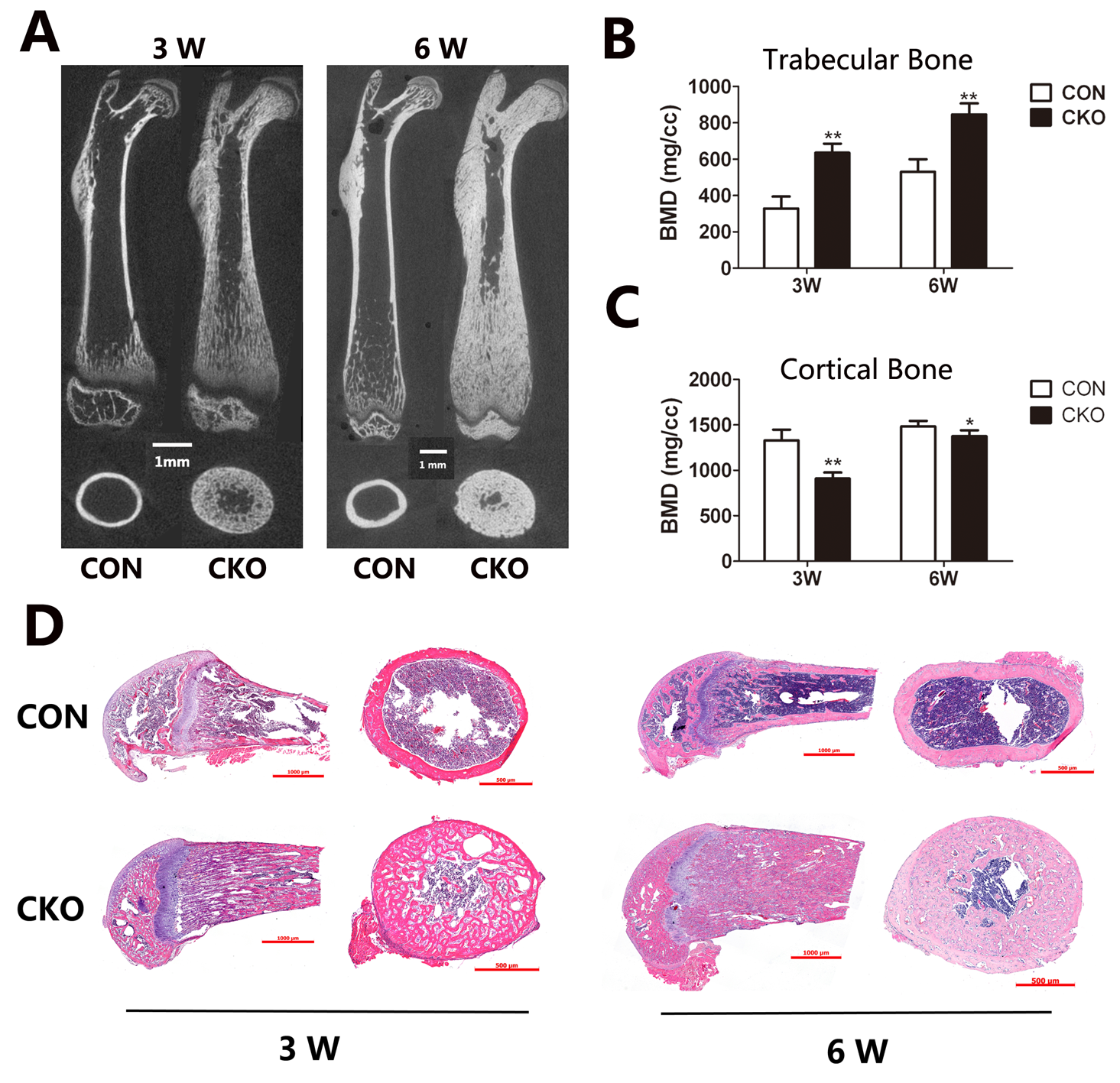

Supplement: Figure S1 — Overproduced trabecular bone in Vhl CKO mice. (A) Representative µCT images of the femurs from OC-Cre: Vhlflox/flox (CKO) and littermate control (CON) mice at 3 and 6 weeks of age. Scale bars: 1.0 mm. (B) BMD of femoral distal metaphyseal trabecular bone of CON and CKO mice at the age of 3 and 6 weeks. (C) BMD of BMD of middle femur cortical bone of CON and CKO mice at the age of 3 and 6 weeks. Data represent mean ± SD. *,p<0.05; **,p<0.01. (D) H&E-stained longitudinal sections of distal femur and cross sections of middle femur from CON and CKO mice at the age of 3 and 6 weeks. Original magnification, ×100. (TIF) [file pone.0099946.s001.tif]

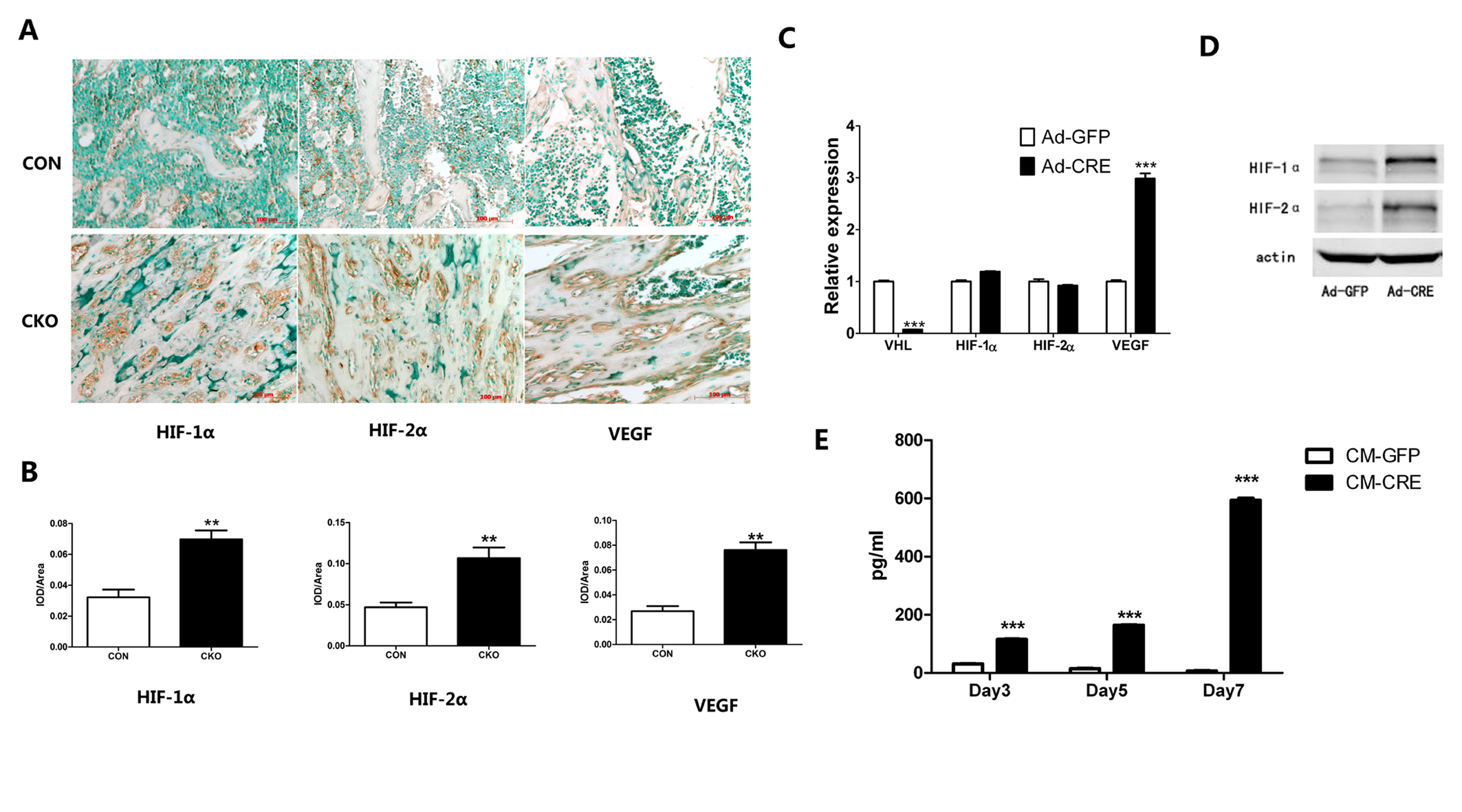

Supplement: Figure S2 — Osteoblasts losing Vhl overexpressed Hif-1α, Hif-2α and Vegf in vivo and in vitro. (A) HIF-1α, HIF-2α, and VEGF protein detection in the distal femoral metaphysis of 3-week-old CON and CKO mice by immunohistochemistry. Original magnification, ×200. (B) Quantitative analysis of A. (C) Quantitative real-time PCR analysis was performed in osteoblasts 48 hours after adenoviral infection. (D) Western blot analysis of HIF-1α, and HIF-2α in osteoblasts. (E) ELISA assay of VEGF (R&D Systems) in the culture supernatant of osteoblasts 3, 5 and 7 days after adenoviral infection. White bars represent Ad-GFP infection; black bars represent Ad-CRE infection. Data represent mean ± SD. *,p<0.05; **,p<0.01, ***,p<0.001. (TIF) [file pone.0099946.s002.tif]
